# Co-occurrence of Antibiotic and Heavy Metal Resistance and Sequence Type Diversity of *Vibrio parahaemolyticus* Isolated From *Penaeus vannamei* at Freshwater Farms, Seawater Farms, and Markets in Zhejiang Province, China

**DOI:** 10.3389/fmicb.2020.01294

**Published:** 2020-06-26

**Authors:** Han Jiang, Ting Yu, Yuting Yang, Shengtao Yu, Jiangchun Wu, Rumeng Lin, Yixian Li, Jiehong Fang, Cheng Zhu

**Affiliations:** Key Laboratory of Marine Food Quality and Hazard Controlling Technology of Zhejiang Province, College of Life Sciences, China Jiliang University, Hangzhou, China

**Keywords:** antibiotic resistance, heavy metal resistance, integrons, *Penaeus vannamei*, sequence type diversity, *Vibrio parahaemolyticus*, virulence genes

## Abstract

*Vibrio parahaemolyticus* is the leading cause of seafood-borne bacterial poisoning in China and is a threat to human health worldwide. The aim of this study was to assess the antibiotic resistance profiles and distribution of heavy metal resistance of *V. parahaemolyticus* isolates from *Penaeus vannamei* from freshwater farms, seawater farms, and their corresponding markets in Zhejiang, China and to assess the relationship between multidrug resistance (MDR) and multi-heavy metal resistance (MHMR). Of the 360 *P. vannamei* samples that we tested, 90 (25.00%) were *V. parahaemolyticus* positive, but the occurrence of pathogenic isolates carrying the toxin genes *tdh* (4.44%) and *trh* (3.33%) was low. None of the tested isolates harbored both the *tdh* and *trh* genes. However, antibiotic resistance profiles varied among different sampling locations, levels of resistance to the antibiotics ampicillin (76.67%) and streptomycin (74.44%) were high overall, and MDR isolates were common (40.00% of all isolates). Heavy metal resistance patterns were similar among the different sampling locations. Overall, the majority of *V. parahaemolyticus* isolates displayed tolerance to Cd^2+^ (60.00%), and fewer were resistant to Cu^2+^ (40.00%), Zn^2+^ (38.89%), Ni^2+^ (24.44%), Cr^3+^ (14.44%), and Co^2+^ (8.89%). In addition, 34.44% (31/90) of isolates tested in this study were found to be MHMR. Using Pearson’s correlation analysis, MDR and MHMR were found to be positively correlated (*P* = 0.004; *R* = 0.759). The 18 *V. parahaemolyticus* isolates that were both MDR and MHMR represented 18 sequence types, of which 12 were novel to the PubMLST database, and displayed a high level of genetic diversity, suggesting that dissemination may be affected by mobile genetic elements via horizontal gene transfer. However, a low percentage of class 1 integrons without gene cassettes and no class 2 or 3 integrons were detected in the 18 MDR and MHMR isolates or in the 90 *V. parahaemolyticus* isolates overall. Thus, we suggest that future research focus on elucidating the mechanisms that lead to a high prevalence of resistance determinants in *V. parahaemolyticus*. The results of this study provide data that will support aquatic animal health management and food safety risk assessments in the aquaculture industry.

## Introduction

*Vibrio parahaemolyticus*, which was first identified in 1950 in Osaka, Japan, is a gram-negative, halophilic, mesophilic, aerobic bacterium that is found naturally in warm marine and estuarine habitats and causes outbreaks worldwide (Baker-Austin et al., 2008; Xie et al., 2017; Yang et al., 2017; He et al., 2019). Some *V. parahaemolyticus* isolates are pathogenic to humans and are responsible for many seafood-related human illnesses, such as gastrointestinal illnesses, diarrheal diseases, wound infections, and even septicemia (Devi et al., 2009; Hu and Chen, 2016; Yu et al., 2016). In addition, according to the official surveillance statistics of the national foodborne disease surveillance system in China, *V. parahaemolyticus* is one of the leading causes of foodborne bacterial poisoning in China (Jiang et al., 2019). The virulence of *V. parahaemolyticus* is attributed mainly to the presence of two genes: the *tdh* gene, which encodes thermostable direct hemolysin (TDH), and the *trh* gene, which encodes thermostable direct-related hemolysin (TRH) (Mok et al., 2019). TDH is a pore-forming, heat-stable protein that remains intact even when heated to 100°C for 10 min. Unlike the thermostable TDH, TRH is heat labile and can be inactivated by heating at 60°C for 10 min (Tan et al., 2017).

The shrimp aquaculture industry accounts for 15% of all internationally traded seafood products (FAO, 2019)^1^. *Penaeus vannamei*, also known as the Pacific whiteleg shrimp, is the most popular shrimp in the world. The production of *P. vannamei* exceeds 70% of total global shrimp production (Peng et al., 2019). It is also one of the most popular shrimp species in China, and China has been the world’s largest producer of *P. vannamei* since 2001 (FAO, 2019)^1^. *P. vannamei* is suitable for aquaculture owing to its fast growth, tolerance to a wide range of water salinity conditions and low temperatures, low dietary protein requirements, and high survival rates (Ghosh, 2018; Xu et al., 2018; Cheng et al., 2019). However, the increasing industrialization of large-scale intensive aquaculture systems and fast-growing shrimp culture models has led to poor hygienic conditions (Yu et al., 2016). As a consequence, the incidence of bacterial infection outbreaks is increasing (He et al., 2016; Yu et al., 2016). Shrimp represents an important reservoir of *V. parahaemolyticus*, especially in fresh and refrigerated stock (He et al., 2019). The distribution of *V. parahaemolyticus* in shrimps from China has previously been reported to be 22–55%, depending on the season, water salinity, and geographic location (Xu et al., 2016; He et al., 2019). The prevalence of *V. parahaemolyticus* is also high in other Asian countries; for example, the rate reported at shrimp farms in India is 35–53% (Silvester et al., 2015; Ananda Raja et al., 2017), and in Malaysia the rate was reported to be 57.8% in retail shrimps (Letchumanan et al., 2015). The use of florfenicol, thiamphenicol, enrofloxacin, flumequine, neomycin, doxycycline, ciprofloxacin, and certain sulfonamides is permitted in the aquaculture industry in China^2^. However, the inappropriate use of antibiotics in aquaculture has contributed to the development of antimicrobial resistant bacteria, imposed serious problems on aquatic ecosystems, and represents a potential threat to human health (He et al., 2016). It has been reported that *V. parahaemolyticus* isolates from seafood and various environments are resistant to a variety of antibiotics, including ampicillin, aminoglycosides, ciprofloxacin, chloramphenicol, and others (Xie et al., 2016; Lopatek et al., 2018). Thus, it is important to monitor variations in the antibiotic resistance profiles of *V. parahaemolyticus* isolates, as they may reveal changes in the sensitivity of the bacteria to antibiotics, particularly first-line treatments of seafood or human infections (Lopatek et al., 2018). In addition, industrial pollution caused by increased industrialization has become one of the most challenging issues facing developing countries (Kang et al., 2018). Among the many industrial pollutants, heavy metals are frequently detected in marine animals and in various environments, such as agricultural soil and rivers (Ansari et al., 2008; Malik and Aleem, 2011). Heavy metals have also been suggested to enhance selection for antibiotic resistance in the environment and vice versa through co- or cross-resistance or coregulation of resistance pathways (Matyar et al., 2008).

The mechanism of coselection is highly favored when diverse resistance genes are located on the same mobile genetic elements (MGEs; Chapman, 2003). Of the various MGEs, class 1 integrons are thought to be strictly correlated to coselection mechanisms, as they are frequently associated with gene cassettes (GCs) in which both antibiotic resistance genes and heavy metal resistance genes are present (Di Cesare et al., 2016). It has been reported that the clinical version of the class 1 integron-integrase gene (*intI1*) has unique advantages as a universal marker of the selective pressures imposed by anthropogenic pollution (Gillings et al., 2015).

Multilocus sequence typing (MLST) is a tool for molecular epidemiology and population genetic studies of bacterial strains that provides consistent typing results of bacterial isolates in different laboratories (Jiang et al., 2019). González-Escalona et al. (2008) developed the first successful MLST protocol for the detection of genetically diverse *V. parahaemolyticus* isolates based on the sequences of internal fragments of seven housekeeping genes (*recA*, *gyrB*, *dnaE*, *dtdS*, *pntA*, *pyrC*, and *tnaA*). Subsequently, many researchers utilized this method to determine the genetic relatedness of global and geographically restricted *V. parahaemolyticus* isolates and to demonstrate the evolution and epidemiology of the bacteria, as this method offers high repeatability (Theethakaew et al., 2013; Urmersbach et al., 2014; Han et al., 2015; Xie et al., 2016; Lopatek et al., 2018; Jiang et al., 2019). MLST also allows for the detection of slowly progressing sequence changes in the *V. parahaemolyticus* genome and may be used to monitor the spread of antibiotic and heavy metal resistance (Lopatek et al., 2018).

Zhejiang, a province in the southeastern coastal region of China, is an important area for *P. vannamei* aquaculture. In cities in Zhejiang that are not located next to the sea, freshwater with low salinity is used for *P. vannamei* aquaculture, whereas seawater is used in coastal cities (Cheng et al., 2019). In this study, we analyzed *V. parahaemolyticus* isolates from *P. vannamei* from freshwater and seawater farms and their corresponding markets in Zhejiang, China to assess their virulence genes and antibiotic and heavy metal resistance profiles. Subsequently, we determined the relationship between multidrug resistance (MDR) and multi-heavy metal resistance (MHMR) of *V. parahaemolyticus* isolates as well as the clonal relatedness of those isolates. Finally, we assessed the role of integrons in the transmission of antibiotic and heavy metal resistance. This information will contribute to the monitoring of the prevalence of antibiotic and heavy metal resistance of *V. parahaemolyticus* isolated from *P. vannamei* and provide insight into the appropriate use of antibiotics and the establishment of risk assessment and health management protocols for aquaculture and seafood consumption.

## Materials and Methods

### *P. vannamei* Sampling

A total of 360 *P. vannamei* samples were collected from a freshwater farm (farm A, *n* = 90), a seawater farm (farm B, *n* = 90), a market where *P. vannamei* cultured at farm A is sold (market A, *n* = 90) and a market where *P. vannamei* cultured at farm B is sold (market B, *n* = 90) in Zhejiang Province, China between 2017 and 2019. Samples were collected from each site during the period from July to September (summer) each year. Each fresh *P. vannamei* sample was placed in a sealed sterile plastic bag (Hope Bio-Technology Co., QingDao, China), transported to the laboratory in a cold box below 4°C, and immediately processed on the day of sampling. The pH value, temperature, and water salinity of each sampling site were recorded using a YSI Professional Plus Instrument (YSI Inc., Yellow Springs, OH, United States).

### Isolation and Identification of *V. parahaemolyticus*

Each fresh *P. vannamei* sample (10–20 g) was mixed with alkaline peptone water (APW; Hope Bio-Technology Co.) containing 3% NaCl (1:1, w/v) in a sealed sterile plastic bag, and the mixture was homogenized for 2 min in a homogenizer (Scientz, Ningbo, China). Homogenates were incubated at 37°C with shaking at 200 rpm for 16–18 h. After incubation, the enriched mixture was streaked onto thiosulfate–citrate–bile salts–sucrose (TCBS, Hope Bio-Technology Co.) agar plates and incubated at 37°C for 16–18 h. Presumptive colonies (green or bluish-green colonies, 2–3 mm in diameter) were selected from each plate and streaked onto chromogenic *Vibrio* agar plates (CHROMagar Microbiology, Paris, France). Because the microbial cells of the aforementioned homogenates were enriched with many of the same colonies, we selected one potential colony (a purple colony, 2–3 mm in diameter) from each sample on the chromogenic *Vibrio* agar plates that represented the characteristics of *V. parahaemolyticus*. The potential colonies were cultured in APW containing 3% NaCl at 37°C for 24 h with shaking at 200 rpm and stored in 20% sterile glycerol at −80°C until further analysis.

### Molecular Identification and Virulence Gene Detection of *V. parahaemolyticus* Isolates

Polymerase chain reaction (PCR) was used to detect the highly conserved species-specific gene *toxR* and the virulence genes *tdh* and *trh* in all *V. parahaemolyticus* isolates (Law et al., 2017). Genomic DNA was extracted using a bacterial DNA extraction kit (Sangon, Shanghai, China) according to the manufacturer’s instructions. The primers used to detect *toxR, tdh* and *trh* are shown in Table 1. Each PCR amplification reaction was performed in a 25 μL mixture containing 250 ng of DNA as the template, 400 nM each primer, 200 mM each deoxynucleotide triphosphate (dNTP), 10 × PCR buffer, and 5 U of Ex-Taq DNA polymerase (Takara-Bio, Beijing, China). PCR amplification was initiated by incubating the reaction mixture at 94°C for 1 min, followed by 30 cycles at 98°C for 30 s, annealing at 55°C for 30 s, and extension at 72°C for 30 s; and a final extension at 72°C for 10 min (Jiang et al., 2019). PCR products (5 μL) were mixed with 1 μL of 6 × loading buffer dye and analyzed by electrophoresis on a 1.2% agarose gel containing GoldView (Sangon, Shanghai, China). *V. parahaemolyticus* isolates ATCC33847 (*tdh*^+^ and *trh*^–^) and ATCC17802 (*tdh*^–^ and *trh*^+^) were used as positive control isolates, and distilled water was used as the negative control.

**TABLE 1:** Polymerase chain reaction primers used in this study.

Target	Primer	Sequence (5′–3′)	Amplicon length (bp)	References
*toxR*	toxRF	TGTTTGGCGTGAGCAAGGTT	340	Primers used in our lab
	toxRR	ATTCACAGCAGAAGCCACAG		
*Trh*	trhF	TTGGCTTCGATATTTTCAGTATCT	500	Silva et al., 2018
	trhR	CATAACAAACATATGCCCATTTCCG		
*Tdh*	tdhF	GGTACTAAATGGTTGACATC	251	Jiang et al., 2019
	tdhR	CCACTACCACTCTCATATGC		
*intI*1	intI1F	GGCTTCGTGATGCCTGCTT	146	Luo et al., 2010
	intI1R	CATTCCTGGCCGTGGTTCT		
*intI*2	intI2L	CACGGATATGCGACAAAAAGGT	789	Odumosu et al., 2013
	intI2R	GTAGCAAACGAGTGACGAAATG		
*intI*3	intI3L	GCCTCCGGCAGCGACTTTCAG	980	Odumosu et al., 2013
	intI3R	ACGGATCTGCCAAACCTGACT		
Cassette arrays in class 1 integron	hep58	TCATGGCTTGTTATGACTGT	Class 1 integron variable region	Odumosu et al., 2013
	hep59	GTAGGGCTTATTATGCACGC		

### Antibiotic Susceptibility Testing

The susceptibility of the confirmed *V. parahaemolyticus* isolates to 18 antibiotics was assessed using the disk diffusion method on Mueller–Hinton (MH) agar (Oxoid Ltd., Basingstoke, United Kingdom) in accordance with the guidelines of the Clinical and Laboratory Standards Institute (Clinical and Laboratory Standards Institute [CLSI], 2017). The 18 antibiotic disks (Hangzhou Microbial Reagent Co., Hangzhou, China) belonged to 8 different classes and were as follows: quinolones (enrofloxacin, 10 μg; ciprofloxacin, 5 μg; norfloxacin, 10 μg), tetracyclines (tetracycline, 30 μg; doxycycline, 30 μg), aminoglycosides (streptomycin, 10 μg), β-lactams (ampicillin, 10 μg; cefazolin, 30 μg; cefamandole, 30 μg; ceftizoxime, 30 μg; cefepime, 30 μg; imipenem, 10 μg), chloramphenicols (chloramphenicol, 30 μg; florfenicol, 30 μg), sulfonamides (trimethoprim–sulfamethoxazole, 23.75 μg/1.25 μg; sulfisoxazole, 300 μg), polypeptides (polymyxin B, 300 μg), and furans (nitrofurantoin, 300 μg). Briefly, fresh cultures were inoculated into LB broth and incubated to a turbidity equivalent to a 0.5 McFarland standard. In a sterile environment, bacterial cultures were placed onto MH agar (Hope Bio-Technology Co.) plates, followed by antibiotic disks. The inoculated MH agar plates were incubated at 37°C for 24 h, and the size of the clear zone of inhibition was used to classify isolates as susceptible, intermediate, or resistant according to Clinical and Laboratory Standards Institute [CLSI] (2017) guidelines. *Escherichia coli* ATCC 25922 was used as a control (Jiang et al., 2019). Isolates resistant to three or more classes of antibiotics were classified as MDR (Cheng et al., 2019).

### Determination of Heavy Metal Resistance of *V. parahaemolyticus* Isolates

To date, no standard method is available to measure bacterial susceptibility to heavy metals (He et al., 2016). According to the method described by Malik and Aleem (2011) with some modifications, the minimum inhibitory concentration (MIC) of heavy metals was determined for each *V. parahaemolyticus* isolate using MH agar containing Zn^2+^, Cu^2+^, Cd^2+^, Ni^2+^, Co^2+^, and Cr^3+^ in varying concentrations (100–3200 μg/mL). Stock solutions of metal salts were prepared in sterilized deionized water and added to MH agar at various concentrations, followed by spot inoculation with approximately 3 × 10^6^ cells. The plates were then incubated at 37°C for 18–24 h. The metals used were ZnCl_2_, CuSO_4_⋅5H_2_O, NiCl_2_, CdCl_2_⋅5H_2_O, CoCl_2_⋅6H_2_O, and CrCl_3_⋅6H_2_O (Shanghai Macklin Biochemical Co., Ltd., Shanghai, China). Isolates were considered resistant if their MIC values exceeded that of the C600 strain of *E. coli* K-12, which was used as a control (Matyar et al., 2008). Isolates resistant to three or more heavy metals were classified as MHMR.

### MLST Analysis

The clonal relatedness of 18 *V. parahaemolyticus* isolates that were both MDR and MHMR were further analyzed by MLST analysis. Seven housekeeping genes (*recA*, *gyrB*, *dnaE*, *dtdS*, *pntA*, *pyrC*, and *tnaA*) were chosen as target genes according to the PubMLST website.^3^ PCR primers, amplification conditions, and sequencing methods are described on the PubMLST website. The sequencing results for each housekeeping gene were analyzed using PubMLST to assign sequence types (STs). If STs or alleles were found to differ from preexisting ones in the database, the strain information of the new STs or the new allelic profiles with forward and reverse trace files were submitted to the database curator to obtain a new serial number (Jiang et al., 2019).

### Detection of Integron Classes and GCs

The presence of integrase genes *intI1*, *intI2*, and *intI3* and GCs was confirmed in all 90 *V. parahaemolyticus* isolates using PCR with specific primers (Table 1). PCR amplification was performed in a 25 μL mixture as described in the foregoing, and reaction conditions included preincubation at 94°C for 1 min, followed by 35 cycles of denaturation at 98°C for 30 s, annealing at 60°C (*intI1*) or 55°C (*intI2* and *intI3*) for 30 s, and elongation at 72°C for 30 s (*intI1*) or 1 min (*intI2* and *intI3*); and a final extension at 72°C for 10 min (Fang et al., 2019).

Because the *intI2* and *intI3* genes were not detected in any of our isolates, the variable regions (VRs) of isolates that were positive for the *intI1* gene were evaluated using PCR. Class 1 integron VRs were amplified using the primers hep58 and hep59 (Table 1) and the following cycling conditions: preincubation at 94°C for 1 min; followed by 35 cycles of denaturation at 98°C for 30 s, annealing at 55°C for 30 s, and elongation at 72°C for 4 min; and a final extension at 72°C for 10 min (Fang et al., 2019).

### Statistical Analysis

All experiments were performed in triplicate. Correlations were identified using Pearson’s correlation analysis. The degree of correlation was considered weak if the correlation coefficient (*R*) was < 0.4, moderate if *R* was between 0.4 and 0.6, and strong if *R* was ≥ 0.6. Differences were considered significant when *P*-values were < 0.05. All analyses were performed using SPSS version 20.0 (IBM Corp., Armonk, NY, United States).

## Results

### Physicochemical Properties of Each Sampling Site

Analytical data for various physicochemical parameters (pH value, temperature, and water salinity) were collected from the four different sampling sites. As shown in Table 2, at the four sampling sites temperature varied from 29.10 ± 0.03 to 32.50 ± 0.05°C, and pH ranged from 6.92 ± 0.03 to 8.00 ± 0.20, which is within the permissible range for shrimp culture. In addition, water salinity varied over a wide range, and was low at farm A (1.75 ± 0.03 ppt) and market A (1.43 ± 0.02 ppt), but was high at farm B (27.10 ± 0.09 ppt) and market B (23.70 ± 0.18 ppt).

**TABLE 2:** Physicochemical parameters of the four sampling sites.

Sources of samples	Temperature (°C)	pH	Salinity (ppt)
Farm A	31.53 ± 0.05	8.00 ± 0.20	1.75 ± 0.03
Farm B	32.50 ± 0.05	7.52 ± 0.12	27.10 ± 0.09
Market A	29.10 ± 0.03	7.64 ± 0.01	1.43 ± 0.02
Market B	30.18 ± 0.09	6.92 ± 0.03	23.70 ± 0.18

### Identification of *V. parahaemolyticus* Isolates and Detection of *tdh* and *trh* Genes

Based on the morphology of colonies on chromogenic *Vibrio* agar plates, 90 (25.00%) isolates from the 360 *P. vannamei* samples were selected for PCR detection. *ToxR*-PCR assays revealed positive amplification of the *toxR* gene, with a 340 bp amplicon band present in 100% of the presumptive *V. parahaemolyticus* isolates, including 3 (3.33%) of the 90 samples from farm A, 31 (34.44%) of the 90 samples from farm B, 20 (22.22%) of the 90 samples from market A, and 36 (40.00%) of the 90 samples from market B. Among these isolates, four from *P. vannamei* at farm B carried the *tdh* gene (4.44%), whereas three isolates from *P. vannamei* at market B carried the *trh* gene (3.33%). None of the tested isolates harbored both the *tdh* and *trh* genes.

### Antibiotic Resistance Profiles of *V. parahaemolyticus* Isolates

The antibiotic resistance profiles of 90 examined *V. parahaemolyticus* isolates to 18 antibiotics from eight classes are shown in Table 3. The profiles varied among the four different sampling locations. Overall, *V. parahaemolyticus* isolates were most resistant to ampicillin and streptomycin, with resistance rates of 76.67% (69/90) and 74.44% (67/90), respectively. In addition, these isolates exhibited relatively high resistance rates for trimethoprim–sulfamethoxazole (64.44%, 58/90), tetracycline (57.78%, 52/90), chloramphenicol (57.78%, 52/90), florfenicol (53.33%, 48/90), enrofloxacin (47.78%, 43/90), sulfisoxazole (47.78%, 43/90), doxycycline (46.67%, 42/90), cefazolin (25.56%, 23/90), and cefamandole (18.89%, 17/90). Low levels of resistance were observed for ceftizoxime (4.44%, 4/90) and cefepime (3.33%, 3/90). None of our isolates demonstrated resistance to ciprofloxacin, norfloxacin, imipenem, polymyxin B, or nitrofurantoin. Furthermore, 40.00% (36/90) of the isolates were classified as MDR.

**TABLE 3:** Prevalence of antimicrobial resistance in *Vibrio parahaemolyticus* isolates.

Antimicrobials		Breakpoint (Clinical and Laboratory Standards Institute [CLSI], 2017)	Percentage of resistant isolates, %				
	
		Resistant/Intermediate/Susceptible (mm)	Farm A (*n* = 3)	Farm B (*n* = 31)	Market A (*n* = 20)	Market B (*n* = 36)	Total (*n* = 90)
Quinolone	Enrofloxacin (10 μg)	≤ 15/16–20/ ≥ 21	33.30	41.94	75.00	38.89	47.78
	Ciprofloxacin (5 μg)	≤ 15/16–20/ ≥ 21	0.00	0.00	0.00	0.00	0.00
	Norfloxacin (10 μg)	≤ 12/13–16/ ≥ 17	0.00	0.00	0.00	0.00	0.00
Tetracycline	Tetracycline (30 μg)	≤ 11/12–14/ ≥ 15	33.33	67.74	65.00	47.22	57.78
	Doxycycline (30 μg)	≤ 10/11–13/ ≥ 14	33.30	67.74	35.00	36.11	46.67
Aminoglycoside	Streptomycin (10 μg)	≤ 11/12–14/ ≥ 15	33.33	74.19	70.00	80.56	74.44
β-Lactam	Ampicillin (10 μg)	≤ 13/14–16/ ≥ 17	66.67	61.29	85.00	86.11	76.67
	Cefazolin (30 μg)	≤ 19/20–22/ ≥ 21	33.30	19.35	35.00	25.00	25.56
	Cefamandole (30 μg)	≤ 14/15–17/ ≥ 18	0.00	25.81	15.00	16.67	18.89
	Ceftizoxime (30 μg)	≤ 21/22–24/ ≥ 25	0.00	6.45	5.00	2.78	4.44
	Cefepime (30 μg)	≤ 18/19–24/ ≥ 25	0.00	3.23	5.00	2.78	3.33
	Imipenem (10 μg)	≤ 13/14–15/ ≥ 16	0.00	0.00	0.00	0.00	0.00
Chloramphenicol	Chloramphenicol (30 μg)	≤ 12/13–17/ ≥ 18	33.33	61.29	45.00	63.89	57.78
	Florfenicol (30 μg)	≤ 12/13–17/ ≥ 18	0.00	67.74	40.00	52.78	53.33
Sulfonamides	Trimethoprim–sulfamethoxazole (23.75/1.25 μg)	≤ 10/11–15/ ≥ 16	66.67	83.87	50.00	55.55	64.44
	Sulfisoxazole (300 μg)	≤ 12/13–16/ ≥ 17	33.33	54.84	50.00	41.67	47.78
Polypeptide	Polymyxin B (300 μg)	≤ 8/9–11/ ≥ 12	0.00	0.00	0.00	0.00	0.00
Furan	Nitrofurantoin (300 μg)	≤ 14/15–16/ ≥ 17	0.00	0.00	0.00	0.00	0.00
MDR			33.33	38.71	40.00	41.67	40.00

### Tolerance of *V. parahaemolyticus* Isolates to Heavy Metals

**FIGURE 1:**
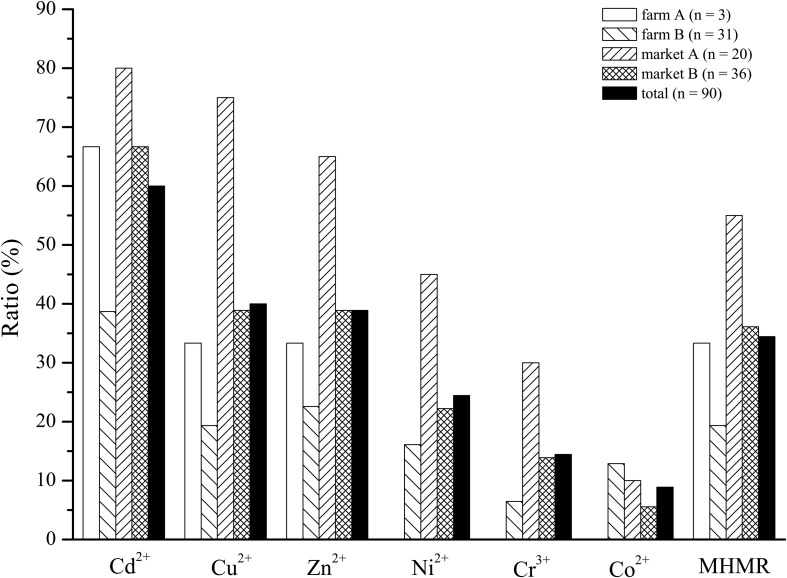
Incidence of heavy metal resistance of *Vibrio parahaemolyticus* isolates derived from four different sampling sites.

In this study, a total of 90 *V. parahaemolyticus* isolates were examined for their susceptibility to heavy metals. When compared to the *E. coli* K-12 C600 quality control strain, maximum MIC values of 3200 μg/mL for Cd^2+^, Cu^2+^, Zn^2+^, and Ni^2+^ and 1600 μg/mL for Cr^3+^ and Co^2+^ were observed (Table 4). Overall, the majority of *V. parahaemolyticus* isolates displayed tolerance to Cd^2+^ (60.00%), and fewer were resistant to Cu^2+^ (40.00%), Zn^2+^ (38.89%), Ni^2+^ (24.44%), Cr^3+^ (14.44%), and Co^2+^ (8.89%). In total, 34.44% (31/90) of isolates in this study were found to be resistant to three or more heavy metals and were classified as MHMR. In addition, as shown in Figure 1, *V. parahaemolyticus* isolates derived from different shrimp sources had similar heavy metal resistance profiles.

**TABLE 4:** Incidence of heavy metal resistance among the *V. parahaemolyticus* isolates.

Heavy metal	MIC (μg/mL)						Resistance	
	100	200	400	800	1600	3200	*n*	(%)
Cd^2+^	5	31^a^	29	17	6	2	54	60.00
Cu^2+^	6	9	10	29^a^	21	15	36	40.00
Zn^2+^	4	19	32^a^	14	18	3	35	38.89
Ni^2+^	10	15	43^ a^	7	13	2	22	24.44
Cr^3+^	0	1	21	55^a^	13	0	13	14.44
Co^2+^	2	80^a^	4	3	1	0	8	8.89
MHMR							31	34.44

*^a^Minimal inhibitory concentration of standard strain E. coli K-12.*

### Relationship Between Pathogenicity, MDR, and MHMR

In our study, although none of the four isolates harboring the *tdh* gene or the three isolates harboring the *trh* gene were MDR or MHMR, our data are insufficient to conclude that there was a correlation between pathogenicity and MDR or between pathogenicity and MHMR. However, MDR and MHMR were found to be positively correlated using Pearson’s correlation analysis (*P* = 0.004; *R* = 0.759).

### ST Diversity

MLST analysis revealed high molecular diversity among the 18 *V. parahaemolyticus* isolates in this study that were both MDR and MHMR (Table 5). A total of 18 different STs were identified, of which 12 (66.67%), namely 2217–2228, were newly identified (shown in bold in Table 5). The numbers of alleles observed for each MLST locus in our study were as follows: 14 *dnaE*, 14 *gyrB*, 11 *recA*, 14 *dtdS*, 10 *pntA*, 13 *pyrC*, and 12 *tnaA*. There were also seven novel loci: *dnaE* 401; *gyrB* 539, 540; *dtdS* 484, 485, 486; and *pntA* 271. All 12 novel STs were submitted to the international PubMLST/*V. parahaemolyticus* database^3^.

**TABLE 5:** Allele profiles, sequence types (STs), virulence genes, integrons, antimicrobial resistance profiles, and heavy metal resistance profiles of 18 multidrug resistant and multi-heavy metal resistant *Vibrio parahaemolyticus* isolates.

Strain	Sources	Allele profiles							STs	Virulence genes		Integrons	Antimicrobial resistance profiles	Heavy metal resistance profiles
		*dnaE*	*gyrB*	*recA*	*dtdS*	*pntA*	*pyrC*	*tnaA*		*tdh*	*trh*			
MA-3	Market A	2	113	28	94	26	83	23	**2217**	-	-	-	ENR-TET-STR-AMP	Zn^2+^, Cu^2+^, Cd^2+^, Ni^2+^
MA-8	Market A	93	224	75	139	117	223	124	919	-	-	-	ENR-TET-STR-AMP	Zn^2+^, Cu^2+^, Cd^2+^, Ni^2+^
MA-17	Market A	47	112	75	97	68	69	64	**2221**	-	-	+	ENR-TET-STR-AMP-CHL-FLO	Zn^2+^, Cu^2+^, Ni^2+^
FB-11	Farm B	76	88	31	13	53	45	13	165	-	-	+	ENR-TET-DOX-AMP-CFZ-ZOX-FEP	Zn^2+^, Cu^2+^, Cd^2+^
FB-15	Farm B	175	22	168	201	130	17	73	471	-	-	-	ENR-STR-AMP-CFZ-ZOX	Zn^2+^, Cu^2+^, Ni^2+^, Cr^2+^
FB-16	Farm B	3	104	144	126	28	226	159	**2219**	-	-	-	TET-DOX-STR-AMP	Zn^2+^, Cu^2+^, Ni^2+^, Cr^2+^
FB-21	Farm B	82	**539**	134	373	26	3	17	**2223**	-	-	-	ENR-TET-AMP-CFZ-ZOX-CHL-SXT-SUL	Zn^2+^, Cu^2+^, Cd^2+^, Ni^2+^, Cr^2+^
FB-23	Farm B	**401**	188	134	**484**	26	3	1	**2224**	-	-	-	ENR-TET-STR-AMP-CFZ-MA-ZOX	Zn^2+^, Cu^2+^, Cd^2+^
FB-31	Farm B	80	**540**	61	**485**	35	141	157	**2225**	-	-	-	ENR-TET-AMP-SUL	Zn^2+^, Cu^2+^, Cr^2+^
MB-2	Market B	5	71	144	**486**	26	11	107	**2222**	-	-	-	ENR-TET-DOX-STR-CHL-FLO	Zn^2+^, Cu^2+^, Cd^2+^, Ni^2+^, Cr^2+^
MB-7	Market B	51	4	25	76	**271**	173	33	**2226**	-	-	-	ENR-TET-AMP-FEP-FLO-SUL	Zn^2+^, Cu^2+^, Ni^2+^, Co^2+^
MB-11	Market B	36	304	188	218	28	168	37	**2220**	-	-	-	ENR-STR-AMP-CHL-FLO	Zn^2+^, Cd^2+^, Co^2+^
MB-14	Market B	175	112	168	201	68	69	64	**2227**	-	-	-	ENR-TET-AMP-CFZ-FLO	Zn^2+^, Cu^2+^, Cd^2+^, Ni^2+^, Cr^2+^
MB-17	Market B	175	22	25	201	130	17	73	**2218**	-	-	-	ENR-TET-AMP-CHL-FLO	Zn^2+^, Cu^2+^, Cd^2+^, Ni^2+^
MB-24	Market B	51	4	168	76	**271**	173	33	**2228**	-	-	-	ENR-TET-STR-AMP-MA-ZOX-FEP	Zn^2+^, Cu^2+^, Cd^2+^, Ni^2+^
MB-29	Market B	175	49	168	201	130	17	73	2174	-	-	-	TET-STR-AMP-MA-ZOX-FEP-CHL-SUL	Zn^2+^, Cu^2+^, Ni^2+^, Co^2+^
MB-32	Market B	153	191	70	19	6	8	1	1160	-	-	-	ENR-TET-STR-AMP-ZOX-SUL	Zn^2+^, Cu^2+^, Cd^2+^, Co^2+^, Cr^2+^
MB-34	Market B	98	49	45	49	4	50	23	1786	-	-	-	TET-DOX-STR-AMP-CFZ-CHL-FLO	Zn^2+^, Cu^2+^, Cd^2+^, Ni^2+^, Co^2+^, Cr^2+^

*Newly identified STs are shown in boldface type. AMP, ampicillin; CFZ, cefazolin; CHL, chloramphenicol; CIP, ciprofloxacin; DOX, doxycycline; ENR, enrofloxacin; FEP, cefepime; FLO, florfenicol; IPM, imipenem; MA, cefamandole; NFX, norfloxacin; NIT, nitrofurantoin; POL, polymyxin B; STR, streptomycin; SUL, sulfisoxazole; SXT, trimethoprim–sulfamethoxazole; TET, tetracycline; ZOX, ceftizoxime.*

### Integron Analysis

Integrons were examined in all 90 *V. parahaemolyticus* isolates. We found that eight isolates (8.89%) harbored class 1 integrons, but no class 2 or 3 integrons were detected. Of the 8 class 1 integron-positive isolates, 4 (11.11%) were recovered from 36 MDR isolates, 3 (9.68%) were recovered from 31 MHMR isolates, and 2 (11.11%) were recovered from 18 MDR and MHMR isolates. No isolates contained GCs. Correlation analysis showed that neither MDR (*P* = 0.550; *R* = 0.192) nor MHMR (*P* = 0.990; *R* = 0.004) was significantly correlated with the carrying of *V. parahaemolyticus* class 1 integrons.

## Discussion

The occurrence of *V. parahaemolyticus* in aquatic samples has raised increasing concern worldwide, as this organism is one of the leading nationwide causes of food-derived bacterial poisoning in humans (Elmahdi et al., 2016; Zhao et al., 2020). In our study, only 3 *V. parahaemolyticus* isolates were detected from 90 samples of freshwater *P. vannamei* cultured at farm A, where the water salinity was as low as 1.75 ± 0.03 ppt. One possible reason for such a low occurrence of *V. parahaemolyticus* isolates is that, within a limited optimal temperature range, water salinity is a driver of *V. parahaemolyticus* levels (Zimmerman et al., 2007). As a halophilic bacterium, the survival of *V. parahaemolyticus* in freshwater ecosystems has been shown to be transient and dependent on the biological host (Nair et al., 2007). Freshwater cultured *P. vannamei* requires multiple gradients to slowly acclimate seedlings to reduced salinity (Peng et al., 2019), and during this process *V. parahaemolyticus* cannot be completely eliminated. However, 20 (22.22%) *V. parahaemolyticus* isolates were found at market A, where freshwater *P. vannamei* cultured at farm A is sold and the water salinity of market A is low, too. Previously, the presence of *V. parahaemolyticus* in freshwater samples from markets was attributed to cross-contamination due to mishandling at fishmongers’ stalls (Nair et al., 2007). Because the surrounding environment of markets is quite complex, some *V. parahaemolyticus* isolates may have been transmitted from the surrounding marine food in the market via human contact, water sources, or other animals. In addition, prevalence rates of *V. parahaemolyticus* were found to be 34.44% at seawater farm B and 40.00% at market B, where *P. vannamei* cultured at farm B is sold, which is consistent with previous studies that assessed marine products in China (Xu et al., 2016; Xie et al., 2017). In our study, water salinity varied over a wide range and was low at farm A and market A but was high at farm B and market B. On the one hand, *P. vannamei* is euryhaline and can tolerate salinity ranging from 1 to 50 ppt (Jaffer et al., 2020). On the other hand, in order to keep *P. vannamei* alive and fresh, water salinity at markets should be similar to that at the farm where the *P. vannamei* was cultured. Similarly, a suitable temperature and pH value should be maintained and an aerator used, as consumers at Asian markets prefer live shrimp to dead shrimp (Xu et al., 2019).

As previously described, the hemolysin markers *tdh* and *trh* play a significant role in the pathogenesis of human infections (Lopatek et al., 2018). In the present study, the *tdh* gene was detected at slightly higher levels, whereas the *trh* gene was detected at lower levels, than those reported in previous studies (Xie et al., 2017; Lopatek et al., 2018). However, other studies reported detecting no virulence genes in aquatic products (Han et al., 2007; Raghunath et al., 2008; Xu et al., 2016). The distribution of *tdh*- and *trh*-positive isolates may vary depending on the sample source, the detection technique, and the geographical origin (Lopatek et al., 2018). It has been reported that clinical isolates have higher rates of virulence genes than isolates from aquatic products, which may be due to environmental factors such as interactions with other hosts and the evolution of pathogens (Letchumanan et al., 2015; Xie et al., 2017). Additionally, *tdh*-positive isolates are more virulent than *trh*-positive isolates (Kang et al., 2018). In our study, none of the isolates from markets A and B were positive for *tdh*, which may represent a reduced risk for serious infections for consumers.

Antibiotic susceptibility testing revealed that *V. parahaemolyticus* isolates were most resistant to ampicillin and streptomycin, with resistance rates of 76.67 and 74.44%, respectively. These results are comparable to data obtained in other countries (Lopatek et al., 2018). It has been reported that the prevalence of ampicillin and streptomycin resistance is very high in *V. parahaemolyticus* from both clinics and aquatic products (Wong et al., 2012; Xie et al., 2017; Silva et al., 2018). This may be due to the extensive use of these antibiotics in aquaculture and antimicrobial residues in aquatic systems (Tan et al., 2017). In addition, compared with gram-positive species, gram-negative bacteria are intrinsically less permeable, which may allow them to resist certain antibiotics, as their outer membrane forms a permeability barrier (Blair et al., 2015).

Tetracycline, ciprofloxacin, chloramphenicol, trimethoprim–sulfamethoxazole, and cephalosporin are first-line drugs used in the clinical treatment of *V. parahaemolyticus* infections and were tested in the present study (Xie et al., 2016; Tan et al., 2017; Yang et al., 2017; Mok et al., 2019). Our findings showed that 57.78, 57.78, and 64.44% of isolates were resistant to tetracycline, chloramphenicol, and trimethoprim–sulfamethoxazole, respectively, which is much higher than rates reported by other studies in several countries and for several sample sources (Yano et al., 2011; Ottaviani et al., 2013; Tan et al., 2017). However, none of our isolates demonstrated resistance to ciprofloxacin, indicating that this antibiotic is still highly effective against *V. parahaemolyticus* and can continue to be recommended as a therapeutic drug. Additionally, *V. parahaemolyticus* isolates in the present study were highly resistant to first- and second-generation cephalosporins (cefazolin, 25.56%; cefamandole, 18.89%); however, fewer than 5% of isolates were resistant to third- and fourth-generation cephalosporins (ceftizoxime, 4.44%; cefepime, 3.33%). This suggests that first- and second-generation cephalosporins may have been misused in the past decades, leading to reduced susceptibility and lower efficiency in the treatment of *V. parahaemolyticus* (Yu et al., 2016). However, the accumulation of third- and fourth-generation cephalosporins in the environment and resulting drug resistance may occur more slowly, and the small number of cases of drug resistance reported in the present study indicates potential future risks. Moreover, high resistance to third-generation cephalosporins has previously been reported in *V. parahaemolyticus* from shrimp in Malaysia, another Asian country (Letchumanan et al., 2019a). In addition, extended-spectrum β-lactamases (ESBLs) confer resistance to a broad range of β-lactams, including third- and fourth-generation cephalosporins, which are commonly found in *Enterobacteriaceae* (Tetens et al., 2019). However, recently, some studies found that the prevalence of ESBL genes varied among new generation β-lactam-resistant *Vibrio* sp. isolates (Bush and Fisher, 2011; Dahanayake et al., 2019). Thus, potential ESBL-mediated cephalosporin resistance mechanisms of *V. parahaemolyticus* should be further researched.

Antibiotics are widely used in aquaculture to control bacterial infections and promote the growth of aquatic organisms. Some of the antibiotics tested in our study, including chloramphenicol, norfloxacin, and nitrofurantoin, have already been banned in food-producing animals in China^4^ ^,5^. In the present study, none of the *V. parahaemolyticus* isolates demonstrated resistance to norfloxacin or nitrofurantoin. Interestingly, we observed high rates of resistance to chloramphenicol (57.78% overall), which has been banned in food-producing animals since 2002. Moreover, a high rate of resistance (53.33% overall) to florfenicol, a fluorinated derivative of chloramphenicol that is widely used to treat aquatic infections in many countries including China, Brazil, and the United States (Yang et al., 2020), was also observed in our study. We found similar results in our previous study of *E. coli* isolates from *P. vannamei* (Cheng et al., 2019). Homology analysis revealed that the sequence of the florfenicol resistance gene *floR* had 55% homology with the sequence of the chloramphenicol resistance gene *cmlA*, both of which are efflux transporters belonging to the major facilitator superfamily (Kadlec et al., 2007; Fang et al., 2019). Thus, we hypothesize that high levels of resistance to chloramphenicol may be related to the use of florfenicol (Yassin et al., 2017). Additionally, although the use of doxycycline, enrofloxacin, and sulfisoxazole is permitted in the aquaculture industry in China^6^, some *V. parahaemolyticus* isolates in our study were resistant to these antibiotics.

Finally, polymyxin B and imipenem are two special antibiotics tested in our study. Polymyxins, including polymyxin B and polymyxin E, have broad-spectrum activity against gram-negative bacteria but are typically considered last-resort antibiotics to treat severe infections caused by MDR isolates (Liu et al., 2016). Imipenem belongs to the carbapenem class of β-lactams, has a very broad spectrum of activity, and acts mostly on gram-negative and gram-positive bacteria (Cheng et al., 2019). Fortunately, none of the *V. parahaemolyticus* isolates in our study exhibited resistance to these two special antibiotics. However, other studies have reported resistance of *Vibrio* sp. to polymyxin B and imipenem. Misuse of polymyxins and carbapenem may have a negative impact on the clinical treatment of *Vibrio* infections in the future (Devi et al., 2009; Lee et al., 2018).

MDR isolates were commonly observed in our study (40.00% overall), which is consistent with findings in previous studies (Xu et al., 2016; Yang et al., 2017). There are many possible reasons for the existence of MDR *V. parahaemolyticus* isolates, including the excessive use of antibiotics for prophylactic use, therapeutic use, or as antimicrobial growth promoters within the aquaculture industry (Xie et al., 2017). The widespread use of antibiotics in clinics, agriculture, and livestock production can result in antibiotic residues entering the environment (Tan et al., 2017), and the exchange of genetic resistance determinants between different environments can occur via direct or indirect contact or via MGEs (Fang et al., 2019). Therefore, non-antibiotic approaches are required to manage the occurrence of antibiotic resistance among *Vibrio* sp. in the environment (Lee et al., 2018). Phages are approved and recognized by US regulatory bodies as potential biocontrol agents to control and inhibit pathogens, including *Vibrio* sp. (Letchumanan et al., 2016, 2019b). Phages pose significant advantages, such as being environmentally friendly and easily discoverable in the environment and having greater host specificity and cost effectiveness than antibiotics (Letchumanan et al., 2016). In addition, some probiotics are possible substitutes for antibiotics against *Vibrio* sp. Tan et al. (2020) identified bioactive compounds with anti-*Vibrio* activity from *Streptomyces* sp. that will be of immense value for the future development of antibacterial agents.

It has been reported that, owing to its unique geographical environment and the rapid expansion of aquacultural, industrial, and agricultural activities, the Zhejiang nearshore area has already suffered heavy metal contamination (Liang et al., 2019). Moreover, heavy metal resistance has been observed in *Vibrio* sp. isolated from aquatic products and the environment in many neighboring provinces of Zhejiang (He et al., 2016, 2019; Hu and Chen, 2016). Some of the heavy metals tested in this study, such as Cu, Zn, Ni, Co, and Cr, are essential micronutrients for bacteria at low concentrations. However, concentrations of such metals above threshold levels, as well as the long-term presence of potentially toxic metals (e.g., Cd in our study), adversely affect the functioning and diversity of microbial communities, which may eventually result in some bacteria developing resistance to heavy metals via complex formation or sequestration of toxic metals, detoxification through reduction of intracellular ions, or extrusion of toxic ions by efflux systems (López-Maury et al., 2002; Matyar et al., 2008; Seiler and Berendonk, 2012; Etesami, 2018). In addition, *V. parahaemolyticus* isolates derived from different shrimp sources had similar heavy metal resistance profiles. The results indicated that the sample source did not appear to greatly impact the heavy metal resistance profiles of *V. parahaemolyticus* isolates, which was also reported in a previous study (He et al., 2016). One possible reason is that industrial pollution may influence the aquaculture environment, as pollutants (e.g., heavy metals) concentrated in the region have a relatively significant impact on certain species and microbes.

In our study, MDR and MHMR were found to be positively correlated. It has been reported that industrial pollutants such as heavy metals may enhance selection for antibiotic resistance and vice versa. One possible reason for this is that metal cations are common environmental stressors that perturb bacteria, activating metal-protective stress responses and growth states that also protect against and provide resistance to antibiotics (Hu and Chen, 2016; Poole, 2017). Additionally, coselection or the coexistence of certain antibiotic resistance genes and heavy metal resistance genes may be beneficial to bacteria for increasing fitness in various environments. Importantly, 18 *V. parahaemolyticus* isolates that were both MDR and MHMR were resistant to Zn^2 +^ and 17 of them were resistant to Cu^2 +^, although their antibiotic and heavy metal resistance profiles were different. Moreover, resistance to ampicillin (94.44%, 17/18), tetracycline (88.89%, 16/18), enrofloxacin (83.33%, 15/18), and streptomycin (66.67%, 12/18) occurred frequently in the 18 isolates. There is a growing body of evidence that Cu/Zn drives antibiotic resistance in metal-exposed bacteria owing to the selection of genetic elements harboring both antibiotic and metal resistance genes and to the recruitment of antibiotic resistance mechanisms by metals. Moreover, Cu^2+^ and Zn^2+^ can bind to certain classes of antibiotics (e.g., the β-lactams, aminoglycosides, tetracyclines, and quinolones tested in this study) to form complexes and hinder antibiotic activity (Poole, 2017). In any case, increases in MHMR and MDR in aquatic products play a crucial role in the food chain and may pose important public health problems (Matyar et al., 2008).

Similar to many previous studies, the STs of the *V. parahaemolyticus* isolates in our study were diverse, and most isolates represented novel STs, indicating a high degree of genomic diversity and suggesting that dissemination of MDR and MHMR genes of *V. parahaemolyticus* isolates from *P. vannamei* samples may be effected by MGEs via horizontal gene transfer (HGT) (Xie et al., 2016; Lopatek et al., 2018; Yang et al., 2017; Jiang et al., 2019). Although they are among the most important and common MGEs implicated in the dissemination and distribution of resistance genes between isolates (Fang et al., 2019), fewer than 10% of all isolates in the present study carried class 1 integrons, and correlation analysis showed that neither MDR nor MHMR were significantly correlated with the carrying of *V. parahaemolyticus* class 1 integrons.

Previous research indicated that the dissemination of antibiotic and heavy metal resistance determinants may be a result of HGT, resulting in the increased prevalence of MGEs in marine aquaculture environments (Rodriguez-Blanco et al., 2012). Among the various MGEs, class 1 integrons are thought to be strictly associated with coselection mechanisms, and harboring the class 1 integron gene can be highly beneficial for bacterial fitness (Gillings et al., 2015; Di Cesare et al., 2016). However, inconsistent with the high prevalence of MDR and MHMR, only a few class 1 integrons were detected among the isolates tested in this study. Similar results have been reported showing that antimicrobial resistance is not related to class 1, 2, or 3 integrons in *Vibrio* sp. isolated from seawater samples in Lima, Peru (Sulca et al., 2018). In addition, other studies have also shown a low prevalence of other MGEs, such as plasmids and integrative and conjugative elements (ICEs), in *V. parahaemolyticus* isolates from seafood in certain provinces in China (Hu and Chen, 2016; He et al., 2019). Thus, *V. parahaemolyticus* in aquatic species may have adopted other molecular mechanisms that mediate the high prevalence of resistance determinants, and this requires further study (Hu and Chen, 2016).

## Conclusion

In conclusion, the results of the present study indicate that *V. parahaemolyticus* occurs in *P. vannamei* from both farms and markets regardless of whether it is freshwater-cultured or seawater-cultured. Few *V. parahaemolyticus* isolates were found to carry the *tdh* (4.44%) or *trh* (3.33%) toxicity genes. However, MDR isolates (40.00%) and MHMR isolates (34.44%) were commonly observed. MDR and MHMR were found to be positively correlated using Pearson’s correlation analysis (*P* = 0.004; *R* = 0.759). Most of the *V. parahaemolyticus* isolates represented new STs, which indicates high diversity among the isolates. However, correlation analysis revealed that neither MDR (*P* = 0.550; *R* = 0.192) nor MHMR (*P* = 0.990; *R* = 0.004) were significantly correlated with class 1 integrons in *V. parahaemolyticus*. Combined with the results of other studies, our findings suggest that *V. parahaemolyticus* in aquatic species may have adopted other molecular mechanisms that lead to the high prevalence of resistance determinants, and future research should focus on elucidating these mechanisms. The results of this study provide data to support aquatic animal health management and food safety risk assessments in the aquaculture industry.

## Data Availability Statement

The datasets generated for this study can be found in the https://pubmlst.org/vparahaemolyticus, 12 novel STs (2217-2228).

## Author Contributions

HJ completed MLST analysis and all data analysis and prepared the manuscript. TY, YY, and SY completed virulence gene detection, antibiotic and heavy metal resistance testing, and integron class and gene cassette detection. JW, RL, and YL completed *P. vannamei* sampling and *V. parahaemolyticus* isolation and identification. JF and CZ designed the project and revised the manuscript. All authors contributed to the article and approved the submitted version.

## Conflict of Interest

The authors declare that the research was conducted in the absence of any commercial or financial relationships that could be construed as a potential conflict of interest.

## Footnotes

1 http://www.fao.org/fishery/static/Yearbook/YB2017_USBcard/navigation/index_intro_e.htm

2 http://www.yyj.moa.gov.cn/gzdt/201909/P020190923348448913072.pdf

3 https://pubmlst.org/vparahaemolyticus/

4 http://jiuban.moa.gov.cn/zwllm/tzgg/gg/201104/t20110422_1976324.htm

5 http://www.moa.gov.cn/govpublic/SYJ/201509/t20150907_4819267.htm

6 http://www.moa.gov.cn/govpublic/SYJ/201008/t20100823_1622639.htm
